# Supplementary figures and images for: Tropane alkaloids and terpenes synthase genes of Datura stramonium (Solanaceae)
Source: PeerJ. 2021 Jun 15;9:e11466. doi: 10.7717/peerj.11466 (PMC8212831; doi:10.7717/peerj.11466)

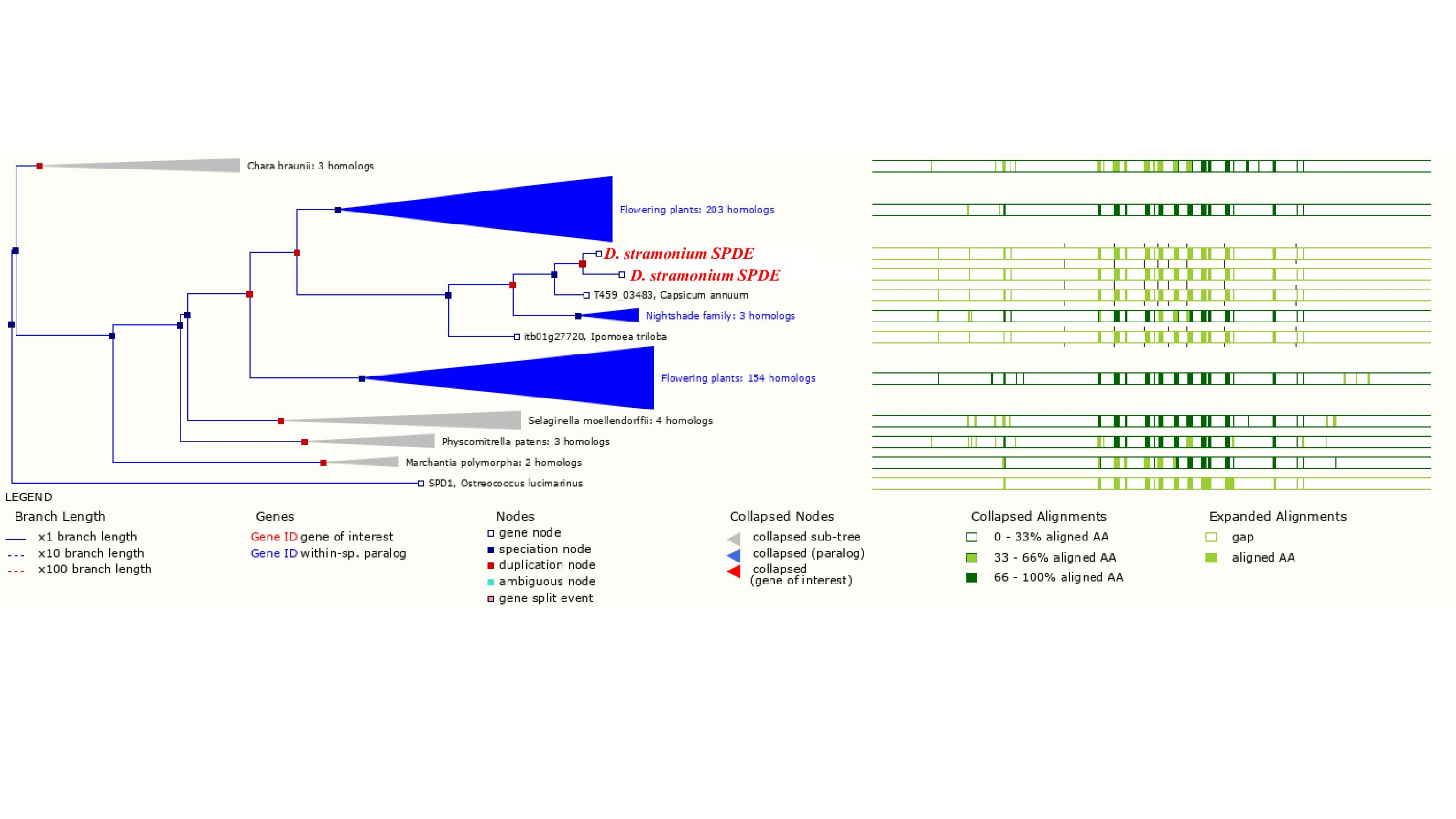

Supplement: Supplemental Information 1 — Phylogeny of PMT in plants. Clades marked with a red square lack duplication events. In the Solanaceae clade, a duplication of Spermidine synthase (SPDE) in Datura stramonium is indicated. [file peerj-09-11466-s001.png]

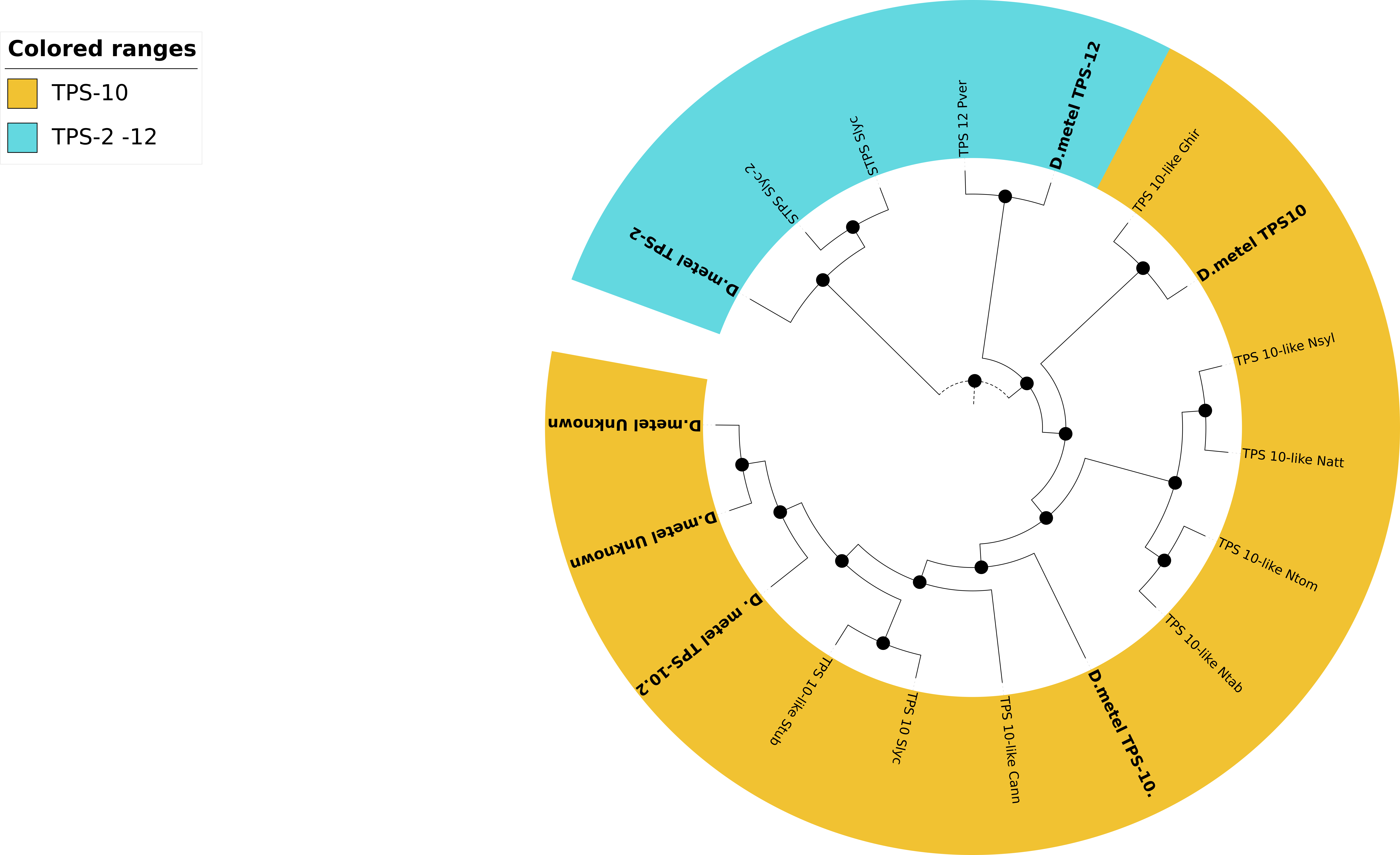

Supplement: Supplemental Information 2 — Bayesian phylogenetic analysis of TPSs found on Datura metel. The seven TPSs found are distributed among Solanaceae in two groups, one represented by TPS-10 (yellow) and the second group, the rest of TPSs (Cyan). [file peerj-09-11466-s002.png]

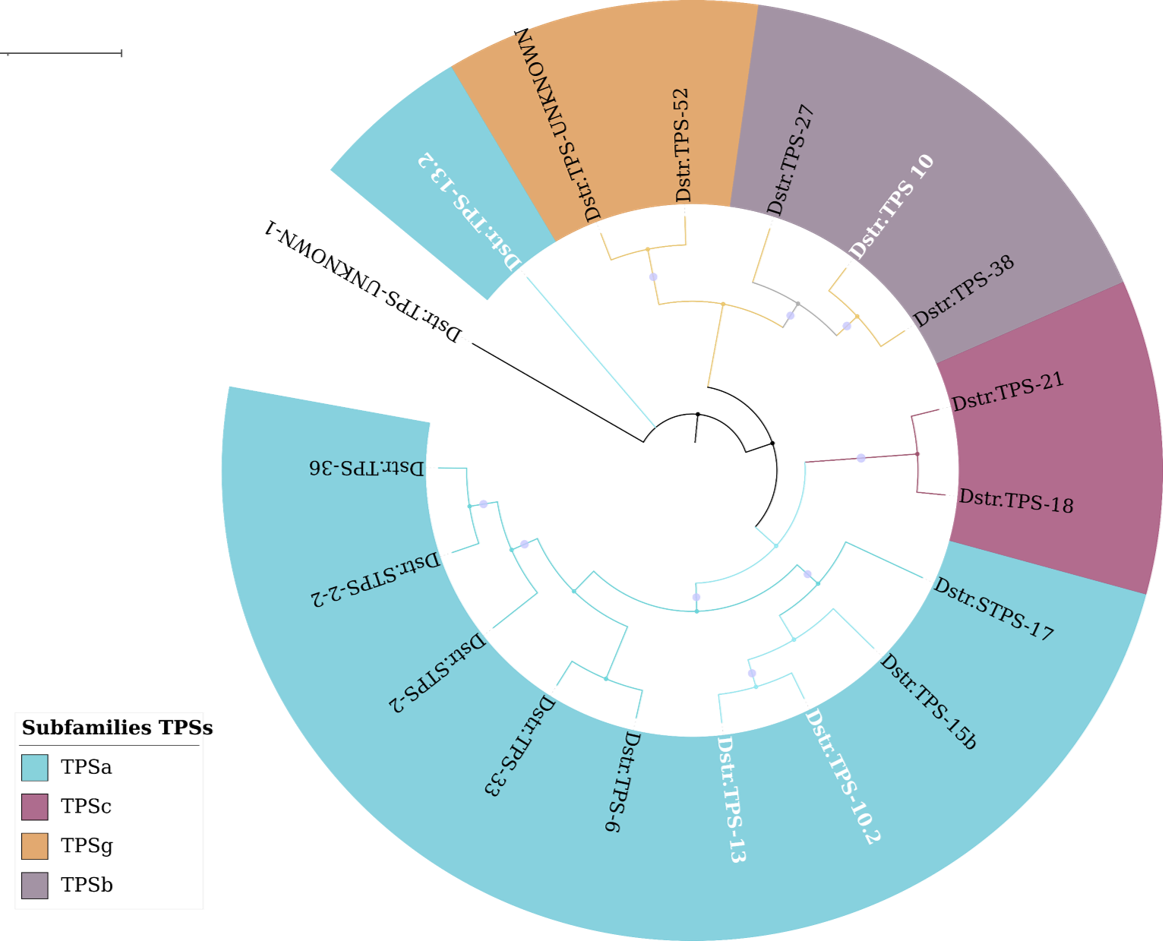

Supplement: Supplemental Information 3 — Distribution of TPSs subfamilies of Datura stramonium. Subfamilies (a, b, c, and g) of the 18 DsTPS found. Nine DsTPS are in subfamily a. Tree obtained by Bayesian inference, with JTT+G+F evolutionary model. Most branches have support above 80%. [file peerj-09-11466-s003.png]

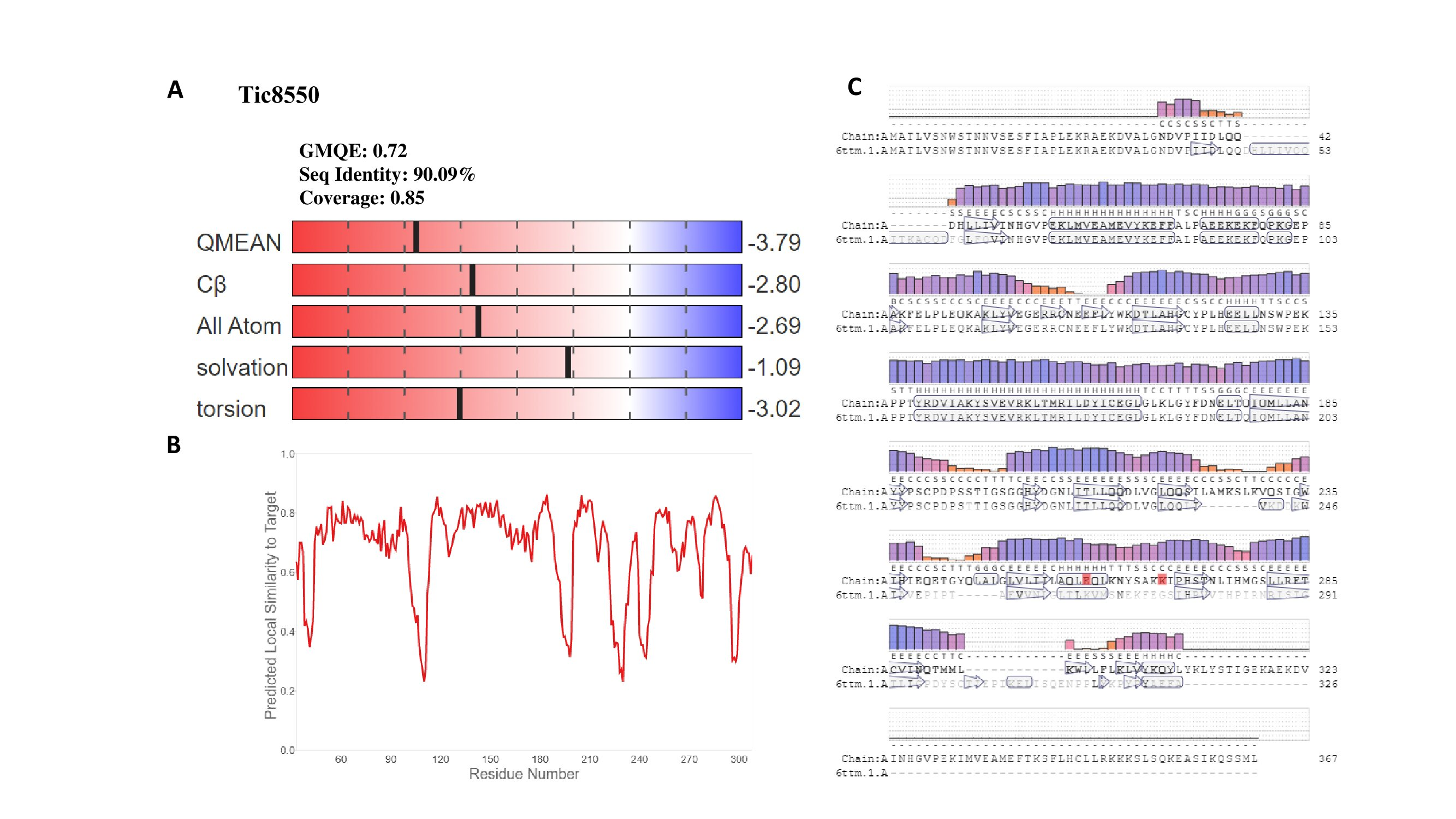

Supplement: Supplemental Information 4 — Quality parameters of modeled protein of Tic8550 (Swiss-Model). (A) Values of GMQE indicate a good precision of construction of protein H6H of Tic8550. (B) For each residue of model x the expected similarity with the native structure y is shown. It is expected that the residues with a punctuation lower than 0.6 being of low quality. In (C) residues present in all polypeptides of proteins of the model are depicted in an interactive screen of protein sequence (H6H). Each residue is labelled with a letter, below the bar graph of the estimated local quality QMEAN), Blue and red color indicate high and low quality, respectively. [file peerj-09-11466-s004.png]

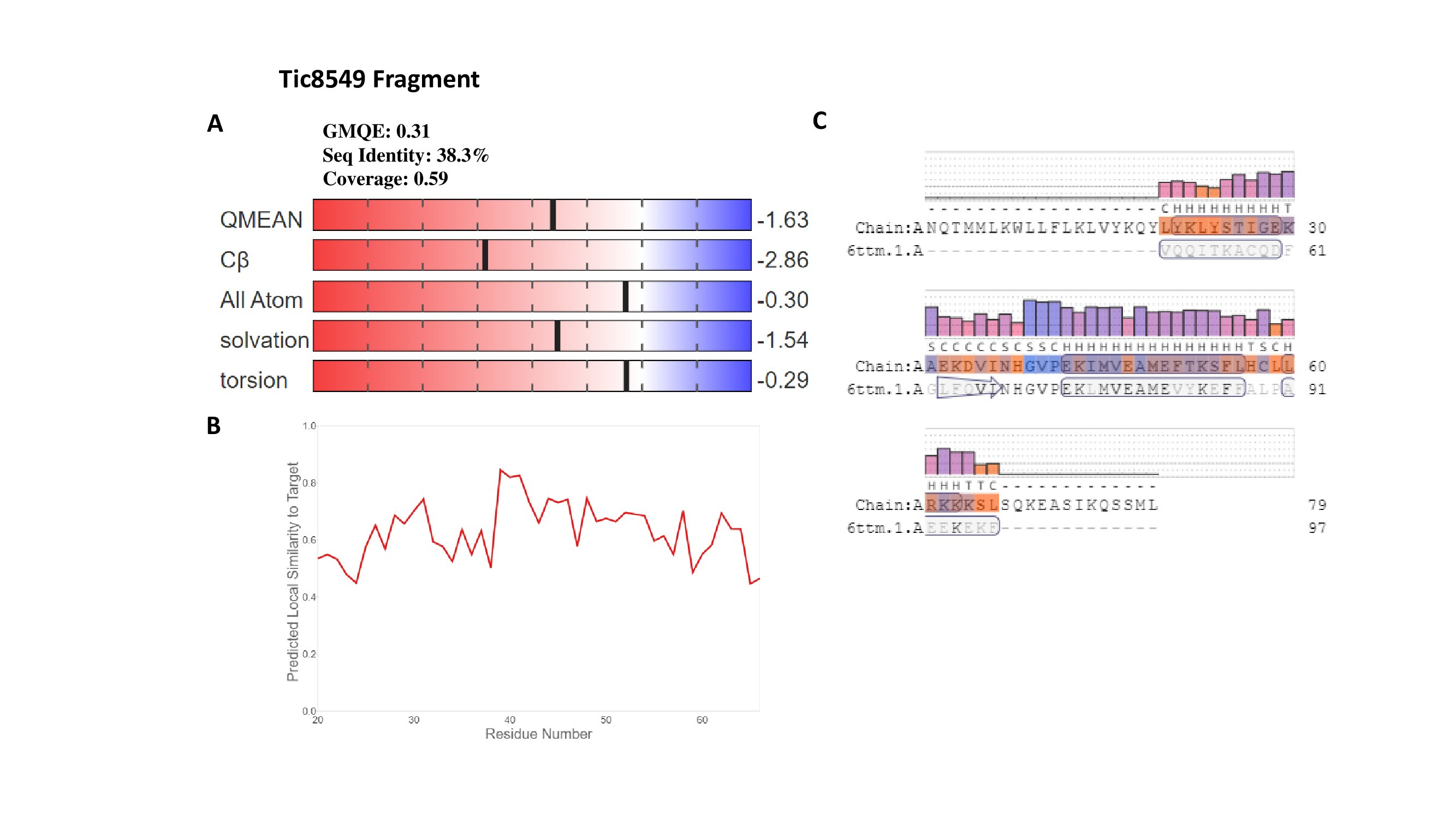

Supplement: Supplemental Information 5 — Quality parameters of modeled protein of Tic8549 (Swiss-Model). (A) Values of GMQE indicate a good precision of construction of protein H6H of Tic8550. (B) For each residue of model x the expected similarity with the native structure y is shown. It is expected that the residues with a punctuation lower than 0.6 being of low quality. In (C) residues present in all polypeptides of proteins of the model are depicted in an interactive screen of protein sequence (H6H). Each residue is labelled with a letter, below the bar graph of the estimated local quality QMEAN), Blue and red color indicate high and low quality, respectively. [file peerj-09-11466-s005.png]

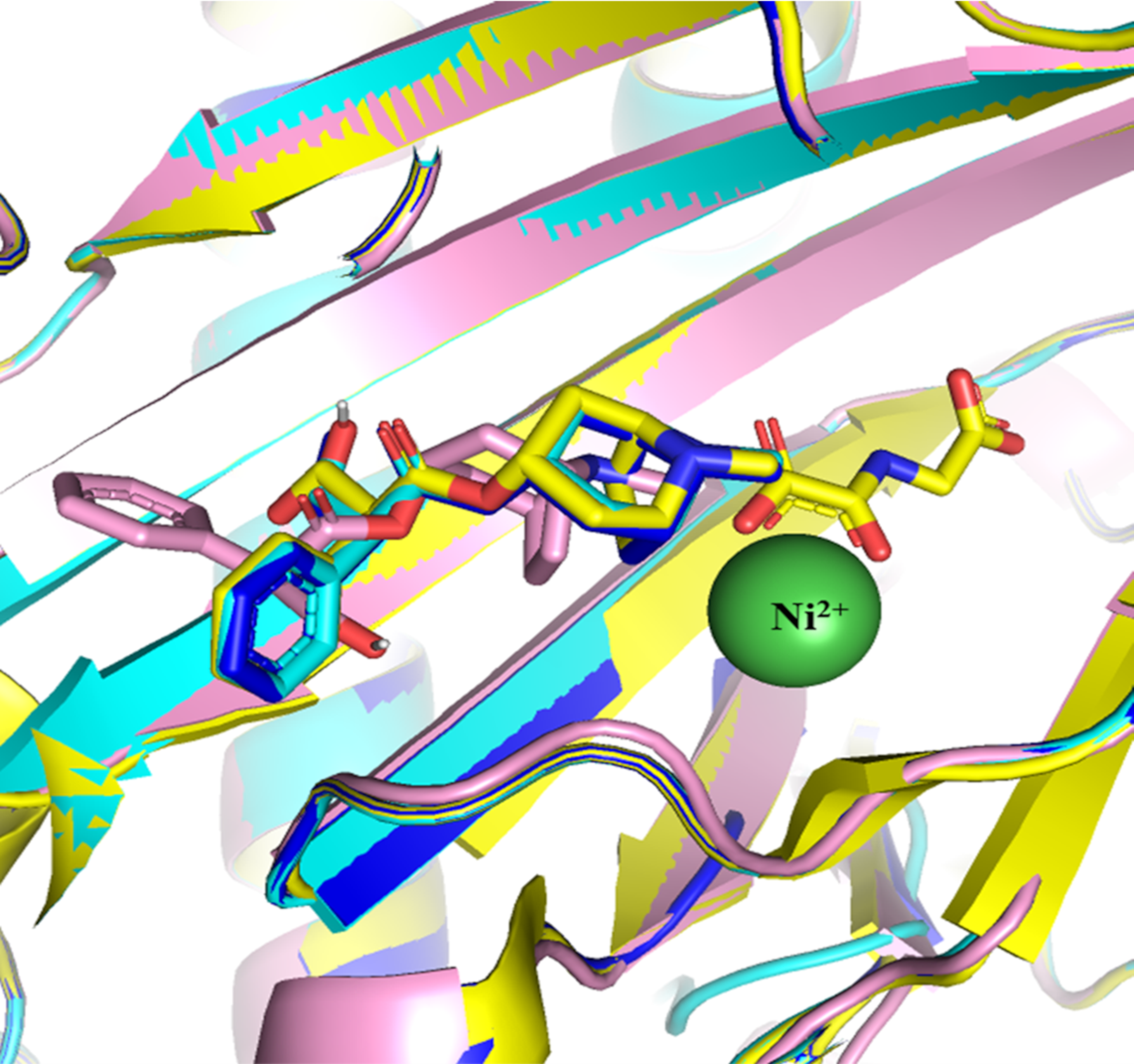

Supplement: Supplemental Information 6 — In silico molecular docking of the native ligand of H6H, which is Hyosciamine (Hy-o), with each of the models and with the crystalized structure of H6H (PDB ID 6TTM). The reference structure 6TTM shows the position of the ligands Hy-o and 2OG and the coordinated metal Ni2+ (in green color). The docking of the H6H structure resulted in a binding affinity (BA) −7.9 Kcal mol−1 and the prediction of the position of Hy (in blue) inside the binding pocket (BP) fits perfectly with the position in the crystalized structure 6TMM (in yellow). The simulation performed with the model Teo8550 produced a BA of −7.1 Kcal mol−1 and the prediction of the position of ligand Hyo fits inside of the BP, but with a different pose. Difference in pose might be due to fact that the structure of the model is slightly different to the model of reference 6TTM. Although the model produced share similar residues inside the BP there are some differences in relation to the reference. [file peerj-09-11466-s006.png]
