# Supplementary material for: Tropane alkaloids and terpenes synthase genes of Datura stramonium (Solanaceae)
Source: PeerJ. 2021 Jun 15;9:e11466. doi: 10.7717/peerj.11466 (PMC8212831; doi:10.7717/peerj.11466)
Supplement: Supplemental Information 7 [file peerj-09-11466-s007.docx]

**Sequences TPS of *Datura stramonium***

>Dstr.TPS-10

MEVKIMLSDQIFDGFYDDSTAGSGENHTAEDTKEMLYLYEAAFLATEGEQGLDASCFGASTALEDVEIRDQVVHQFLQDKIRQCDPLLASLDFNIVQAANLQDLKYVATWWKETGLAENLPFARDRLVENYFWTIGVNFLPEYGYFRRIATKVNALVTPIDDIYDVFGTLHELQIFTDAIQRWNMDELDKLPDNMKMCYFALDNFINEVGCDAFEEQGIFILPYLRNAWRGLCKSYLREAKWYYSQDMPTMEEYMDNAWISISAPVILAHAYFLVANPVNNEALHYLENNYHDIIRCSALILRLANDLGTSSDEKFIRRRDELKMEVKIMLSDSNMKQLEQLEIIDYLQRLGLSYHFEDEIYSILNIRDKNSKRDHLYAKALEFKLLRQHGFNISQEIFDGLCDDSTAGCGEIHTTKDTKGMLYLYEASFLAIKGEKELDAPCFGASTALEDVEIRDQVVYQFLQEKTRQYHPFLLELASLDFNIVQAAHLQDLKYVATWWKETGLAENLPFARDRLVENYFWTIGVNFLPEYGYFRRIATKVNALVTTIDDVYDVFGTLHELQIFTDAIQRWNIDELDKLPVPDNMKMCYFALDNFINEVASDAFEEQGIFILPYLRNAWRDLCKSYLREKWYYSQYMPTMEEYMDNAWISISAPVILVHAYFLVANPVNNEALHYLENNYHDIIRCSALILRLANDLGTSSEELKRGDVPKSIQCYMNESKCSEEEARQHIRVLISQTWKKLNEAHDIAAHPFPKIFVTCAMNLARMAQCMYQHGDGHGGNNSTTKNRITALLFESIPLISEYGPEGK

>Dstr.TPS-10-2

IFILPYLRNAWRGLCKSYLREAKWYYSQDMPTMEEYMDNAWISISAPVILAHAYFLVANPVNNEALHYLENNYHDIIRCSALILRLANDLGTSSDEKFIRRRDELKMEVKIMLSDSNMKQLEQLEIIDYLQRLGLSYHFEDEIYSILNNIMQQGQVFSQKDLNLRQRLLRQHGFNISQEILVTTIDDVYDVFGTLHELQIFTDAIQRWNIDELDKLPVPDNMKMCYFALMELLKDYDIAIFEEQGIFILPYLRNAWRDLCKSLYHPGKANMVADALSSKIVSMGNPVNNEALHYLENNSLLAWRLLNGQWPGKSELKRGDVPKSIQCYMNESKCSEEEARQILWKKAAAHPFPKIFVTCAMNLARMAQCMYQHGDGHGGNNSTTKNRITALLFESIPLISEYGPEGK

>Dstr.TPS21

MILGYRSKIISPSDHKLIRNGKIGSSTWVAMVPSRHSPKQPCFPQCLDWILENQREDGSWGLNPSHPLLVKDSLSSTLACLLALHKWRLGDKQVQRGIGFIETCYGWAVDNKDQISPLGFEIIFGSMIKYAEKLNLNLPLHSDFINLAICKRDSTIESVEYMAEGLGELCQWEEIMLHQRHNGSLFDSPATTAAALIYHQHNEHCFEYLNSILQLHKYWEEKTEFADEEHLFASRSGKFTSDVGILELHKASQLAIHEKDHILDKINNWTAIFMHQKLLNNDFLDMNSNKEFYTTYDRVENRRYIKSYEENKFKMLKTAYRSPSINNKDLLIFSTHDFDLCQTQHREELQQLKRWYEDCRLDRLGLSEQYIYTTYLIGVAVVFEPEFSDARLMYAKYVMLLTVIDDLFDGFASKDELLNIIELVERWDDYASVGYNSERVKVFFSVFHKTIEELAAIAEIKQGRSVKDHLTNLWIEVMKCMLIERLEWWTSKTRPSIEEYLHVTCVTFGSRLIPLTTQYYLGIKLSKDLLESDEICGLCNCTGIVMRLLNDLQTYKSIYEEEAIRQIKEILESNRRKLLKMVLVQRKGSQLPQLCKDIFWRTSKMVYFTYSHGDEYRFPEEVKNHIDQVIYKPLNH

>Dstr.TPS-36

MGVAYHFDNEIETFLQNIFDASSQQNEINDDNLYVVALRFRLVRQQGHYMSSDVFEQFTDHDGKFKKTLINDVQGLLSLYEAAHLSVHGEEILEEALAFTITHLESMVPILTKSLKVQVTEALSHPVHRTIPRVVTGKYINIYENMESHNHLLLKFAKLDFNMLQKLHHRELSELTRWWKDVDLANKLPYARDKLVESYFWTLGVYFEPQYSRARKLLVKVFNMISTNDDTYDAYATLNELVLYTDAIQKWDISAMDSLPPYMRPLYQVNLDIFSEMEEELAKEGNSERIYYGTFEMKKLLRSYFEDAKWYDAGYIPKCEEYIKNALVSSAIMTLSTNSLVGMERFIAKDIFEWVMNEPLIVRASSAICRLMDDIIDHEVQQERGHAASIIECYMKEYGASKEEAYTKYRKEVKNAWKDINKALMRPTEVPMFVLERPLNLARLLDTLFKDEDGYTNSHTKCKDLITMLLTESFDI

>Dstr.TPS-15b

M-AGFG-AYHFENEIEESLNNIYIN-GYEEWIGEFGESDLHPVALSFRLLRQQGVYFEPQYKVARNILTKVLCFISITDDIYDTYGTLHELTLFTNAIERWNIDASESLPSYMKFVYVVLLDFYSEVERELTKKNNSFRVKYSIDAMKKLVRSYFEEAKWYNGNKVPTMEEYVKNGIPSSSTPCLAIASLLGMGNNEATKEAFDWIATEPPILVASSIIARLLNDIVSHERELERGDVASGIECYMNEYGATKEEAYLEMKKIIENKWKDLNREYLKRINLPRIWLMPVINLARIAEFVYKDEDAYTVSKNNLRVIISMVLVDPITI

>Dstr.TPS-33

MAPTAVMSTYEEEEIVRPVADFSPSLWGDSFHSFSIDNQVAEIYAQEIETLKKQTRSMLSAACETTSAEQLNLIDTIERLGIAYHFEKQIEDILDQIYKANPNFEAHDLCTLSLQFRLLRQHGYNISPKILSRFQDANGKFKESLSNDIRGLLNLYEASHVRTHGEDILEEALAFTTAHLESAAPHLKSPLRKQVTHALEQSLHKSIPRVETRYFISIYEEEESKNDVLLRFAKLDFNLLQMLHKQELSEVSRWWKDLDFVTTLPYARDRAVECYFWTVGVYVERQYSQARIMLAKTIAMISIVDDTFDAYGIVKELEVYTDAIQRWDISQIDRLPDYMKISYKALLDLYNDYEIELSTDGRSDVVQYAKERMKEIVRNYFVEAKWFIEGYMPPVYEYLSNALATSTYYLLTTTSYLGMKCANKEDFEWLATNPKILEANVSLCRVIDDIATYEVEKGRGQIATGIECYMRDYGVSTKEAMEKFQEMAETAWKDVNEGILRPTPVSTEILTRILNLSRIIDVTYKHNQDGYTHPEKVLKPHIIALLVDSIEI

>Dstr.TPS-13

MRSLRLARNHSTSTYERLVYEGWILYDTGHREEALAKAEESISLQRSFEAFFLKAYALADTTLDSESSTYVIQLLEEALKCPSDGLRKGQALNNLGSIYVDCNKLDLAADCYVSALEIKHTRAHQGLARVYHLKNDRKSAYEEMTKLMDKAQNKASAYEKRSEYCDRDMANSDLGMATALDPLRTYPYRYRAAVLMDDQRETEAVEELTRAISFKPDLQMLNLRAAFHESMTDFSRALQDSEAALCLDPNHKDTLDLYSRTHIWLRAAQHSDVHGLMT

>Dstr.TPS-13-2

MSTSTDNESEGKQQSCEERNISKSRFVSLIRQCSLELSHTMRGFKLKDRCKTTQVHAYNPSETSTSNPFPIAATSKLHYPTINSILAESPDSVSPSTAESFLPYGLPRTDFFEPPVELCLKSVDFVESLAELYRKVQMTQDFNKYLVHLEQYALLYSLGDPKLLRRCLRSARQHAVDVHSKVVLSAWLRFERREDELVGSSALDCIGRVLECPKAALIHGYDPNSVFDHCECHGAANEISDFGTSDGNNFLSSEKGGIVYFCVGNEEVNCIRGKIAALSGPLKSMLYGDFIESDKERIDFTHIGISVDGMRAVDFFSRTRRLGSYPPHILLELLSFANRFCCEEMKSASDCYLASLVSDIDEALVLIDYALEERAHLLVASCLQLMLRELPGYLYNPKVLNTFCSSEARERLATVGQASFLLYYFLSQVAIEDNMMSKVTVMLLERLKDCASERWQKALASHQLGCVLLERKDYKEAQHSFEMAIEAGHVYSVVGVARTKFKQGQRFLAFELINGIISEYTPTGWMYQERSLYSLGKQKILDVNDATRLDPTLSFPYKYRAIVMVEESKIEAAIVEINRIVGFKVSSDCLELRAWCFIALEDYQSAIRDIQALLTLEPNYMMFCGKMRADHLVELLSLHVQPWSPADCWMQLYNRWSSVDDIGSLAVIHQMLINDPGRSILRFRQSLLLLR

>Dstr.STPS-2

MAASPNNSRPLANFHSTVWGYHFLSYTPELTEITPQEKVEVDELKERIRKMLVETPDESTQKLVLIDTIQRLGVAYHFHKEIETSQNIFDASQQQSENDNNDDNLYVVALRFRLLRQQGHYMSSDVFKKFTDEDGKFKETLTNDVRELLSLYEAAHLRVHEEEILDEALSFTTGHLEFMLSDSSNDSLKVQITEALSEPIHKTVSRMGARKYISIYENNDAHDNLLLKFAKLDFNVLQKLHQRELSELTRWWKYLNFANKLPYVRDRLAECYFWILGMYFEPQYNRARRMMTKVLKLSSIIDDTYDAYGKLDELASFTDAIQRWDVSAIDSVASYLKPVYQVLLDVYSEMEQVLAKECNSYRVYYAKYEMKKLVRAYFKEAQWLSAGYIPNCEEHMENALVSCGCLMAATTSLAGVEEFISKETFEWLMNEPLIFESSSILCKSNGRYYWT

>Dstr.gSTPS-6

MTSLAGNVNNFSDFRPEANFSPSLWGNIFSYSTQDTQVSEKIIEEIQTLKEEVKHMIISSTTSNDTREKIHLIDTLERLGISYHFKEDIEDQLSKMFDLNVIHDEDDLYNVALYFRLFRQHGYPISSDHFNQFKDNNKFKETLLEDAKGLLSLYEAAHVRKHGDDILEEALIFATSHLEKITPTLDSTLEKQVRHALMQSLHRGIPRAEAHFNISIYEECGSENEKLLRLAKLDYNLLQMLHKEELRELTQWWKDLNFASKLSYVRDRMVECFFWTVGVYFEPQYSRARVMLAKCITMISVIDDTYDSYGTLNELVVFTDAVDRWDVSEVDRLPNYMKMIYTSLLDLFKEYETEIKEQDRFSGVYYVKEAMKEIVRSYYIEAEWFIEGKIPSFKEYLSNALVTGTYYLLAPASLLGMRSASKEAFDWMMNKPKILVSSAIIGRVIDDIATYKIEKEKGQLVTGIECYMQENNLSIEEASAELSEIAENAWKDLNKECIKPTNMSSEILMRVVNLARLIDVVYKNNQDGYSNPKNNVKSVIEDLLVNPINM

>Dstr.TPS

MALFEDLYGRRCRSPIGCFEVGEAAMIGPDSVHEAMVKVQLIRDRLKTTQSRQKSYADVKRRELEFVVDDWVFLKVSPMKGVMRFERTEKLVPAMLVPIGL

>Dstr.STPS-17

MELCTQTVAADHKVITRRTADHHPTVWGDHFLAYADILVGANYEGEEKQHEGLKEEVRKMLGNKLAPSKSLEKLELINIIQRLGVAYHFEREMEKLLSYFYSRYEEEWIVDSNLHAVALCFRLLRQQGYYVSSDVFRKFTDDQGDFKKTLVNDVQGMLSLYEAAQFRVHGEQILDEAANFTTTQLKLILPKLSNSLAQQVSSALKFPIRDGIVRVETRKYISIYKEDESCNEVLLKFAKLDFNILQRLHKKELCDITRWWKELEIMKELPYARDRLVELYFWSLGIYFEPQYSVARKIVTKVLC-FCSIMDDTYDTYGTLDELTLFADAIERWNIDASEKLPPYMKIIYRALLDVYNEIEQELANENKSFLVNYSINEMKKLSRAYFQEAKWYHGKNVPTVEQYVKNGGLSSTYHLIAASFLLGMEEVATKDAFDWIATEPLILVASGVIARLLNDIVSHEIEQERGDVASGIECYMNEYGVTKQEARMEMRKIIENCWKNINQEYLRPTIVISRVLLMLVINLTRVSEFIYKDEDAYSFSKNNLKDVISTVLIDPIIIT

>Dstr.TPS-27

MQEGKYKREAQKLKEEVLCLVAEIGNPLAKLELTDSINKMALSHLFDKEIMVSLQDMEYANKKDSGTEMDLYSTALHFRIFRQYGYNVPQDVFRSYMDEMGEQFKVDADMDPNTMMQLFEASHLALKDENMLDEARIFCTKNLKSSNDIMPLHWKVEWYNTTRQISKQENEERGPLNSKLLELAKLNFNMVQAEHQKDLIDILRWWRKLELIENISFSRDRIVESFLWSVGVAFEPQHSRVRSWLTKAITFIIVIDDVYDIYGSLQDLEIFTNAVVRWDPKEVEQLPSCMKLCFRKLYDTTNDIALAIQQQKGWKLPVSTYLQKVWAEFCKALLVEAKWDSKGYTPTFCDYLDNGWKSSGGTVLSLHVLLGLAQHFSQVNDFLENQQHLIYYSSLIIRLCNDLGTSTAELERGDVASCILCYMSKENVKEDVAREHIKGMVMETWKKMNKHCFHN-SSSLGPLMKYIMNMARVAHFIYQ-YGDGFGVQDRETRQQILSSLVESLPLN

>Dstr.TPS-38

MDVRRSGNYKPSIWEDGYVQSRANLYSEEKYCERAEELKQEVRRILEKTMTHSLEQLEFIDILQRLGIYYHFEEEIDRVLKQIYVNYNKTDHHNGLKNEELYATALEFRLLRQHGYHAPQEIFCSFMNEEGKFKTSLVEDTKGLLSLYEASYLSVEGENIMDKARDFATHCLMESLRKKMDQNLAEQVSHALEMPLHWRMERLEQRWFIQVYHEKVTNMNPVLLELAKLDYNMVQATYLEELKQMSRWDKNIKLVKRMNFVRDRLVEGFFWAVGFTPNPQFGYCRKLSTKLSVLLTTIDDIYDVYGTLDELELFTDIVDRWDINAIEQLPEYMKICFLALFNSMNELAYDVLKEQGFSIISHIRKEGMKWYQRRYTPSLNEFLRNAWITNTGPVLIMHAYFCITNPIKEEELECLKHYPSIIYSPSLILRLVNDLATSPDEIKKGDYLKSIQCYMHESKSSEENARNYIKSLIDQTWKKMNRDILRDRSFSKDFRRTAMNLARIAQCIYQHGDGFGIPDRETKDRILSLFFEPIPLS

>Dstr.TPS.18

MVRTRATAAVEQEPVLEAGSTTKAWVAMVPSRHSLKQPCFPQCLDWILENQREDGSWGLNPSHPLLVKDSLSSTLACLLALHKWRLGDKQVQRLGFIEMHGWAIDNKDQISPLGFEIIFPSMIKSAEKLNLNLPLDSTIKRTLRNNFTWSTIGNLEYMAEGFGELFPTMYPMKMHSLLSLVDTLQSLGIHRYFKSEIKEVLDETYRLWQQKDEEIFSNVANCAMAFRLLRMSNYEVSSEEIAEFADEEHFFARSGKFTSDVGIIELHKASQLAIHEKDHILDKINNWTAIFMQKKTLKQ

>Dstr.TPS-52

MVGFDSEREITRRCANHHPTIWGDHFLTYANLLGANEWEEKKHEDLKEEVRKMLIMSPSKSLQNLDLINTIQLLGVDYHFEHEIEESLSNIFNGYEEWIGEFDESDLHVVALIFRLLRQQGYYVSSDIFRRFTDEQGNYKKSLVSDVQGLLSLYEAVQFRVEGEEILDEAVNFTTTHLKQILPNLSDSFAMQVSNALKFPINSNIVRMKKLLRAYFQEAKWYHGMTVPTMEQYIKNGIPSSTYLLLATTSWLGMGDVATKEAFDWISSEPTILVSSCIIARLLNDLVTHEVVYLILQIELERGDVASGIECYMNEFGVTKKEAYMEIRKIIENNWKDLNRGCLKP-TTVPRVLLMPVLNLTRVAEFFYKDEMLILFLKTT

>Dstr.STPS-2-1

MRAPFERPRRPWVGNYVDPPVRVGIRHSKKWHAIRSRTKMGWSIPHIRLISIAVQFTLLCACARGTNYRYKHSYSLQYRSPPCSDTVIYIRGHSRSWRPRGRVSNYVVYIQLCSICFAYSVSVVLTPVWGAALLACSTGRPGPCPDHLISILRGLQIVYIYLPVVSCAGRVGPCEISSQEKYNHEELKEKVREKLVETPDYSTQKLVLIDTIQRLGVAYHFKNEIKTSVGNIFDEFEQSENKDDDDDLYVVALRFRIVRQQRHYMSSDVFKKFTNDDGKFKETLTKDVPGLLSLYEAAHLRVHGEEILEEALTFTIAHLESMGPKLGNLLKAQVSEALIRPIHTNVPRVGASKYLRIYENIESHDDLLLKFAKLDFNILQKGHQGELCELTRWSIDLDLVNKYPYARDKLVECYFWAVGMYFEPQYRHARRILTKLVAMITLTDDLYDAYATYDELVPFTNAVER

>Dstr.TPS_UNKNOWN

MTWKFMLVLAWLTTLICSTTCSSRGGGSYVRDACRVTRYQDLCIHSLASFSSTAKDNPSKWARAAVSVTISQAKSVTQSLLELRKHNFLKGRGRIAILDCVDCLQDALDNLHNSLGVLRKLSSQTFNDQMGDVTTWLSTALTDEDTCLDGLEDKKRKQVKLLLNRVTNVSYMTSNALALVNKLATNGPQCLENS
