# Supplementary material for: Tropane alkaloids and terpenes synthase genes of Datura stramonium (Solanaceae)
Source: PeerJ. 2021 Jun 15;9:e11466. doi: 10.7717/peerj.11466 (PMC8212831; doi:10.7717/peerj.11466)
Supplement: Supplemental Information 8 [file peerj-09-11466-s008.docx]

**Protein sequences of TPS extracted from the transcriptome of *Datura metel***

>D. met_TPS_2

MELCTQTVAADHKVITRRTADHHPTVWGDHFLAYADILVGANYEGEEKQHEGLKEEVRKMLGNKLAPSKSLEKLELINIIQRLGVAYHFEREMEKLLSYLYSRYEEEWIVDSNLHAVALCFRLLRQQGYYVSSDVFRKFTDDQGDFNKTLVNDVQGMLSLYEAAQFRVHGEQILDEAANFTTTQLKLILPKLSNSLAQQVSSALKFPIRDGIVRVETRKYISIYKEDESCNEVLLKFAKLDFNILQRLHKKELCDITRWWKELEIMKEFPYARDRLAELYFWTLGVYFEPQYSVARKIVTKVLCFCNIMDDTYDTYGTLDELTLFADAIERWNIDASEKLPPYMKIIYRALLDVYNEIEQELANENKSFLVNYSINEMKKLSRAYFQEAKWYHGKNVPTVEQYVKNGGLSSTYHLIAASFLLGMEEVATKDAFDWIATEPLILVASGVIARLLNDIVSHEIEQERGDVASGIECYMNEYGVTKQEARMEMRKIIENCWKNINQEYLKPTIVISRVLLMVVINLTRVSEFIYKDEDAYSFSKNNLKDVISTVLIDPIIIT

>D.met_TPS_10_LIKE

VMATIISASIISKPILIWTLQVPKSCNISNAQAAPLFWTIGVNFLPEYGYFRRIATKVNALITTIDDVYDVFGTLHELQIFTDAIQRWNIDELDKLPLPDNMKMCYFALDNFINEVGCDAFEEQGIFILPYLRNAWRDLCKSYLREAKWYYSQYMPTMEEYENAWISISAPVILVHAYFLVANPVNNEALHYLENNYHDIIRCSALILRLANDLGTSSEELKRGDVPKSIQCYMNETKCSEEEARQHIRVLIGQTWKKLNEAHDIAAHPFPKIFVTCAMNLARMAQCMYQHGDGHGGNNSTTKNRIMALLFESIPLISEYGPEGK

>D.met_TPS_10_LIKE_2

VMATIISASIISKPILIWTLQVPKSCNISNAQAAPLSLEQLEVIDYLQRLGLSYHFEDEIYSILNNIRDKNSKKDYLCAKALEFRLLRQHGFNISLEIFDGLCDASTAGCGEIHTTKDTKGMLYLYEASFLAIKGEKELELARNLTEKHLREYLAGNKNNYMDQSLVELVHHALELPLHWRMLRLETKWFINFYKKRQDNIIPFLLELA

>D.met_TPS_10

AKALEFRLLRQHGFNISLEIFDGLCDASTAGCGEIHTTKDTKGMLYLYEASFLAIKGEKELELARNLTEKHLREYLAGNKNNYMDQSLVELVHHALELPLHWRMLRLETKWFINFYKKRQDNIIPFLLELFWTIGVNFLPEYGYFRRIATKVNALITTIDDVYDVFGTLHELQIFTDAIQRWNIDELDKLPLPDNMKMCYFALDNFINEVGCDAFEEQGIFILPYLRNAWRDLCKSYLREAKWYYSQYMPTMEEY

>D.met_TPS_12

YICDLFKRARLSDNRTADTPLETNARYS

>D.met_TPS_10_LIKE_1

TSASVLTLPEGTDGFVVYSDASRIKLGCMLMQHGKVIAYASGQLKVHEKNYPTHDLELAAVVFTSKIWRHYKNGGLSSTYHLIAASFLLGMEEVATKDAFDWIATEPLILVASGVIARLLNDIVSHEIEQERGDVASGIECYMNEYGVTKQEARMEMRKIIENCWKNINQEYLKPTIVISRVLLMVVINLTRVSEFIYKDEDAYSFSKNNLKDVISTVLIDPIIIT-

>D.met_TPS10-LIKE_3

NAWISISAPVILVHAYFLVANPVNNEALHYLENNYHDIIRCSALILRLANDLGTSSEELKRGDVPKSIQCYMNETKCSEEEARQHIRVLIGQTWKKLNEAHDIAAHPFPKIFVTCAMNLARMAQCMYQHGDGHGGNNSTTKNRIMALLFESIPLISEENAWISISAPVILVHAYFLVANPVNNEALHYLENNYHDIIRCSALILRLANDLGTSSEELKRGDVPKSIQCYMNETKCSEEEARQHIRVLIGQTWKKLNEAHDIAAHPFPKIFVTCAMNLARMAQCMYQHGDGHGGNNSTTKNRIMALLFESIPLISEYGPEGK
